# *spa* Typing and Antibiotic Resistance Profiles of Methicillin‐Resistant *Staphylococcus aureus* Isolates From Broilers in Ilam, Iran

**DOI:** 10.1002/vms3.70976

**Published:** 2026-04-28

**Authors:** Zeinab Alimadad, Fazel Pourahmad, Mostafa Nemati

**Affiliations:** ^1^ Department of Microbiology, Faculty of Veterinary Sciences Ilam University Ilam Iran

**Keywords:** antibiotic resistance, methicillin‐resistant *Staphylococcus aureus* (MRSA), *spa* typing, poultry

## Abstract

**Background:**

Methicillin‐resistant *Staphylococcus aureus* (MRSA) poses a significant threat to public health and the poultry industry due to its resistance to β‐lactam antibiotics and its potential for transmission between animals and humans.

**Objectives:**

This study aimed to characterize MRSA isolates from broilers in Ilam, Iran, utilizing *spa* typing and evaluating their antibiotic resistance profiles.

**Methods:**

A total of 200 samples were collected from nasal and cloacal swabs of broilers. Bacterial isolation and biochemical tests were conducted. The presence of *S. aureus* was confirmed through polymerase chain reaction (PCR) targeting the *femA* gene. PCR amplification of the *mec*A gene was performed to identify MRSA. Additionally, the *spa* gene was amplified in all identified MRSA strains and in one representative strain of methicillin‐sensitive *Staphylococcus aureus* (MSSA). Antibiotic susceptibility testing was conducted on all 107 *S. aureus* isolates.

**Results:**

A total of 237 bacterial strains were isolated. Of the 107 confirmed *S. aureus* isolates, 9 (8.41%) were identified as MRSA. *spa* typing revealed five distinct types, including four for MRSA and one for MSSA. Notably, three novel *spa* types (t304, t567 and t1184) were reported for the first time in Iran, reflecting unique local adaptations, with t011 (40%) being the most common.

**Conclusions:**

This study underscores the genetic diversity of MRSA in poultry and highlights the urgent need for effective control measures to combat antibiotic resistance.

## Graphical abstract

This study characterized MRSA in broilers in Ilam, Iran. Of 200 swabs, 107 *Staphylococcus aureus* isolates were confirmed by *femA* PCR, of which 9 (8.41%) were MRSA (*mecA*‐positive). *spa* typing revealed five distinct types, with t011 dominant (40%). Novel types for Iran included t304, t567 and t1184. Antibiotic resistance profiling highlighted public health risks. Findings confirm significant MRSA genetic diversity in local poultry, underscoring the need for control measures to prevent resistance transmission.

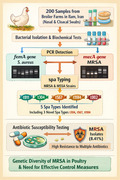

## 1 Introduction

The increasing prevalence of methicillin‐resistant *Staphylococcus aureus* (MRSA) in food‐producing animals, particularly poultry, has significant public health implications. Poultry is a major source of protein worldwide; however, the misuse of antibiotics in the industry promotes the emergence of resistant pathogens, thereby increasing the risk of zoonotic transmission (Gan et al. 2021). MRSA resistance arises primarily through the *mecA* gene, which encodes penicillin‐binding protein 2a (PBP2a), conferring resistance to β‐lactam antibiotics (Lakhundi and Zhang 2018). The emergence of MRSA in community settings, often linked to food animals, raises concerns about its transmission to humans (Zhang et al. 2023). MRSA is a well‐documented pathogen that causes severe infections in humans and animals (Aedo and Tomasz 2016). It has significantly impacted global public health due to its ability to evade treatment by resisting β‐lactam antibiotics, mainly through the expression of the *mecA* gene. This gene encodes PBP2a, which prevents β‐lactam antibiotics from binding effectively, making them ineffective. MRSA is categorized into healthcare‐associated (HA‐MRSA), community‐associated (CA‐MRSA) and livestock‐associated (LA‐MRSA) based on its source and epidemiology (Gopal and Divya 2017; El‐Hamid Abd et al. 2019). The emergence of MRSA in food‐producing animals, including poultry, is a significant concern as it connects animal health to human health through zoonotic transmission. Poultry farms, which are crucial to global food production, have been increasingly identified as reservoirs for MRSA (Anjum et al. 2019; Hossain et al. 2022; Khairullah et al. 2024). Several studies report that broilers carry MRSA in their nasal cavities, cloacae and other body parts, leading to contamination during slaughtering and processing. As a result, MRSA can enter the food chain, posing risks to consumers (Nacer et al. 2024; Stewart‐Johnson et al. 2019). The role of antibiotics in poultry farming is twofold: as therapeutic agents and as growth promoters. However, their overuse and misuse have significantly contributed to the development of resistant bacteria, including MRSA (Rizqullah et al. 2025). In Iran, poultry farming is a major agricultural activity, making it a critical area for understanding MRSA dynamics in the region. Poultry farming represents a cornerstone of Iran's agricultural economy and food security framework, supporting a population exceeding 89 million. With annual production surpassing 2.5 million metric tons of poultry meat and over 1.2 million tons of eggs, the sector accounts for roughly 15%–20% of the nation's agricultural GDP and employs more than 1 million individuals directly and indirectly (Shariatmadari 2000; Javadi et al. 2024). This industry not only provides an affordable, high‐quality protein source—critical for addressing micronutrient deficiencies in low‐ and middle‐income settings—but also bolsters export revenues, contributing to non‐oil economic diversification (Seyedeh Mozhgan et al. 2025). Amid rising population pressures and climate challenges, poultry's scalability underscores its vital role in mitigating food insecurity, with per capita consumption reaching 28 kg of meat annually, far outpacing other meats (Rahimi and Karimi 2015). Surveillance for zoonotic pathogens like *S. aureus* in this sector is thus imperative to safeguard public health and sustain productivity. Molecular epidemiology methods, especially *spa* typing, are essential for understanding MRSA diversity and monitoring its spread. The *spa* gene of *S. aureus* encodes protein A, a surface protein that facilitates immune evasion through its interaction with host immunoglobulins. This gene features a polymorphic X region comprising variable repeat sequences, which serve as molecular markers for strain classification, particularly among MRSA isolates. *spa* typing, which specifically targets this hypervariable domain, represents a reliable and cost‐effective approach for molecular epidemiology, with enhanced discriminatory power when integrated with antimicrobial susceptibility testing (Harmsen et al. 2003). This region comprises 24–26 amino acid repeats of varying lengths (typically 21–27 bp), flanked by conserved regions suitable for polymerase chain reaction (PCR) amplification and Sanger sequencing (Mellmann et al. 2007). Repeats are assigned unique numerical codes based on sequence identity, and the resulting *spa* type is defined by the ordered string of these codes (e.g., t011: 04‐17‐20‐17‐23‐17‐34‐17‐16‐02‐16‐16‐17‐34‐16‐16‐16‐17). This single‐locus sequence‐based method offers high resolution for outbreak investigations and clonal tracking, outperforming multilocus sequence typing in cost and speed while correlating strongly with phylogenetic clades (Krukowski et al. 2020).

Its global popularity stems from the centralized Ridom SpaServer database, which curates over 22,489 *spa* types from 179 countries and 470,913 strains, enabling real‐time epidemiological inference (El‐Ghany 2021). Adopted in surveillance networks across Europe, North America and Asia, *spa* typing has illuminated zoonotic transmissions, including from livestock to humans. In poultry‐associated *S. aureus*, it has revealed livestock‐adapted clones, underscoring its relevance for One Health approaches (Cuny et al. 2022). Despite the global importance of MRSA in poultry, data from Iran remain scarce. This study aimed to address this gap by characterizing MRSA isolates from broilers in Ilam Province through *spa* typing and assessing their antimicrobial resistance profiles. Understanding the genetic diversity and resistance patterns of MRSA in this setting is crucial for developing effective intervention strategies and reducing risks to both animal and human health.

## 2 Materials and Methods

### 2.1 Sample Collection

Two hundred samples were collected from nasal and cloacal swabs of 100 apparently healthy live broiler chickens (five birds per farm) originating from 20 commercial broiler farms across five counties in Ilam Province, Iran. Chickens were sampled at a commercial slaughterhouse upon arrival from these farms, which were purposively selected in collaboration with local veterinary services to represent geographical diversity within the poultry production region of Ilam Province (spanning ∼20,000 km^2^). Each farm housed 10,000–20,000 birds sourced from common Iranian hatcheries. Flocks showed no clinical signs of disease (morbidity <5% in the preceding week), and routine antibiotic use (e.g., tetracyclines and sulphonamides) had ceased at least 14 days prior to sampling. Swabs were taken from birds at 35–42 days of age during pre‐slaughter health assessments, prioritizing random selection to capture baseline carriage without bias toward symptomatic individuals. Samples were placed in sterile transport medium, stored at 4°C, and transported within 2 h to the Microbiology Research Laboratory at the Faculty of Veterinary Sciences, Ilam University, for analysis. This slaughterhouse‐based approach minimizes transport‐related artefacts and aligns with protocols for detecting subclinical *S. aureus* in poultry production chains.

### 2.2 Bacterial Isolation and Identification

Samples were cultured on blood agar and mannitol salt agar at 37°C for 24–48 h. Colonies characteristic of *S. aureus* were subjected to catalase and oxidase biochemical tests. Confirmed isolates were preserved in tryptic soy broth with 20% glycerol at −20°C for further testing.

### 2.3 Antibiotic Susceptibility Testing

The Kirby–Bauer disk diffusion method was used to determine resistance profiles on Mueller–Hinton agar with a bacterial suspension of 0.5 McFarland turbidity standards, following the Clinical and Laboratory Standards Institute (CLSI 2021) guidelines (Mangaraj and Barkataki 2017).

### 2.4 Molecular Characterization

DNA extraction was performed using a boiling method on isolates confirmed as *S. aureus* following biochemical tests (Kobayashi et al. 1994). PCR was conducted to detect the *femA* gene (Abdullah and Hassoni 2024; Vannuffel et al. 1995). The *mecA* gene was investigated in all confirmed *S. aureus* isolates as described previously (Al‐Talib et al. 2014). All methicillin‐resistant (*mecA* gene‐positive) isolates, along with a representative isolate of methicillin‐sensitive *S. aureus* (MSSA) (*mecA* gene‐negative), were subjected to *spa* gene amplification using a PCR assay (Kahl et al. 2005). The list of PCR primers used in the study is indicated in Table 1.

**TABLE 1:** List of oligonucleotides used in the study.

Target gene	Primer sequence (5′–3′)	Product size (bp)	Reference
*fem*A	F‐CGA TCC ATA TTT ACC ATA TCA R‐ATC ACG CTC TTC GTT TAG TT	450	Seyedeh Mozhgan et al. (2025)
*mec*A	F‐AAA ATC GAT GGT AAA GGT TGG C R‐AGT TCT GCA GTA CCG GAT TTG C	533	Rahimi and Karimi (2015)
*spa*	F‐TAA AGA CGA TCC TTC GGT GAG R‐CAG CAG TAG TGC CGT TTG CTT	Variable	Kahl et al. (2005)

### 2.5 *spa* Typing

Amplified *spa* gene products were purified using a commercial PCR purification kit (Yekta Tajhiz, Iran) and sent to Macrogen, South Korea, for Sanger sequencing. The resulting sequences were analysed for *spa* typing using the Ridom SpaServer online tool, which classified the sequences based on established *spa* type designations.

## 3 Results

### 3.1 Biochemical Tests

**FIGURE 1:**
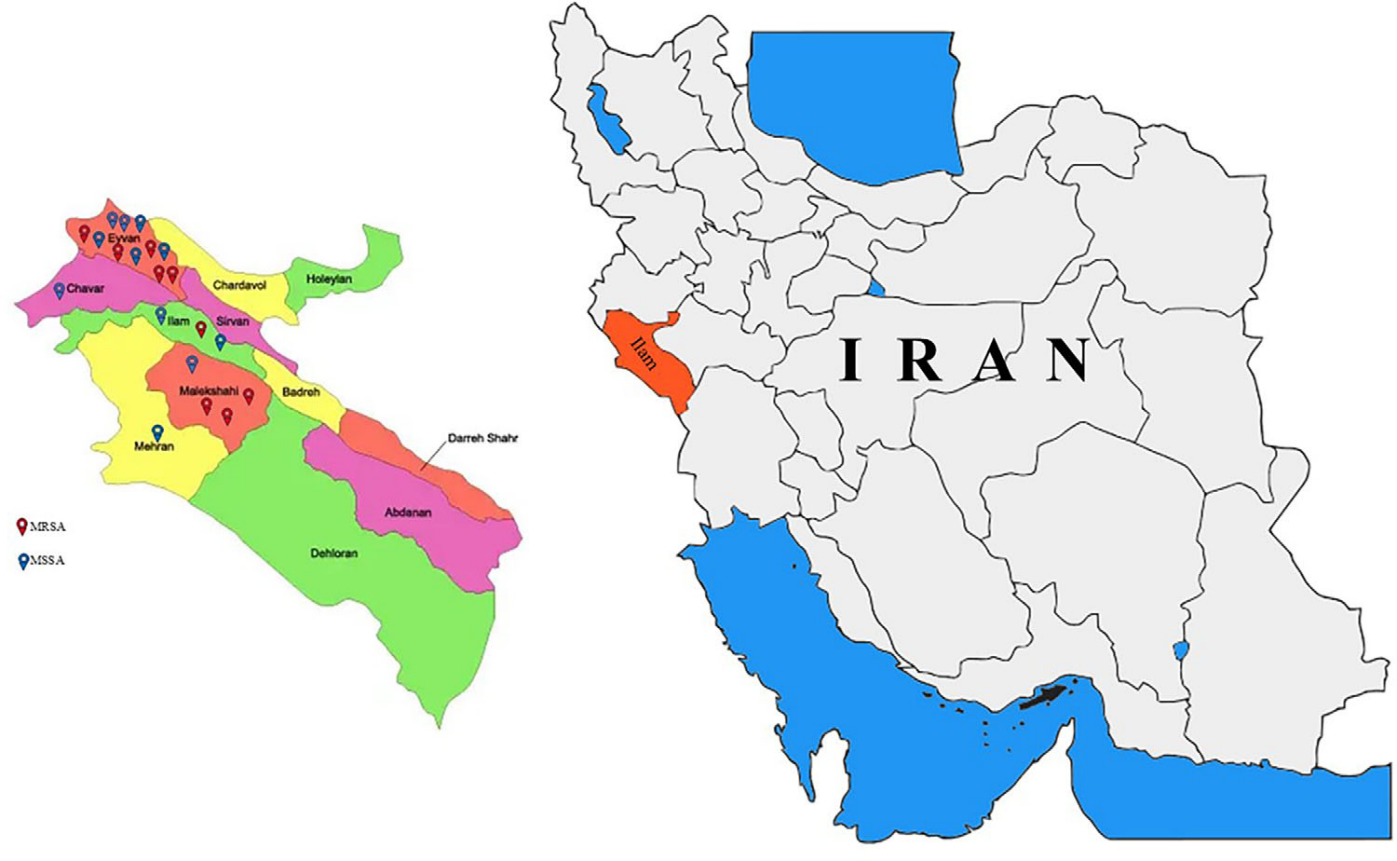
Geographic distribution of sampled poultry farms in Ilam Province, Iran. Farms with MRSA isolates are marked with red pins, whereas farms with MSSA isolates are marked with blue pins. MRSA, methicillin‐resistant *Staphylococcus aureus*; MSSA, methicillin‐sensitive *Staphylococcus aureus*.

The geographical location of the studied farms is presented in Figure 1. A total of 237 isolates were recovered from nasal and cloacal swabs collected from the studied farms. Of these, 122 isolates (51.5%) were biochemically confirmed as *S. aureus*. The farm‐wise distribution of the recovered isolates and the biochemically confirmed *S. aureus* isolates is summarized in Table 2. Among the confirmed *S. aureus* isolates, 73 (59.83%) originated from nasal swabs, whereas 49 (40.16%) were obtained from cloacal swabs.

**TABLE 2:** Farm‐wise distribution of *Staphylococcus aureus*, *femA*‐positive and *mecA*‐positive (MRSA) isolates in Ilam province.

Farm ID	Samples (*n*)	City	*S. aureus* (biochemical tests)	*S. aureus* (*femA*)	MRSA (*mecA*)
A	10	Eyvan	9	7	1
B	10	Eyvan	7	6	1
C	10	Ilam	5	4	1
D	10	Eyvan	7	6	1
E	10	Malekshahi	8	7	1
F	10	Malekshahi	9	7	1
G	10	Malekshahi	7	6	1
H	10	Malekshahi	6	6	0
I	10	Eyvan	6	5	1
J	10	Eyvan	4	4	0
K	10	Eyvan	6	6	0
L	10	Eyvan	5	4	1
M	10	Eyvan	6	6	0
N	10	Eyvan	4	3	0
O	10	Chavar	6	5	0
P	10	Eyvan	4	4	0
Q	10	Mehran	7	6	0
R	10	Eyvan	5	5	0
S	10	Ilam	6	6	0
T	10	Ilam	5	4	0
**Total**	**200**	**5**	**122**	**107**	**9**

### 3.2 Molecular Analyses

**FIGURE 2:**
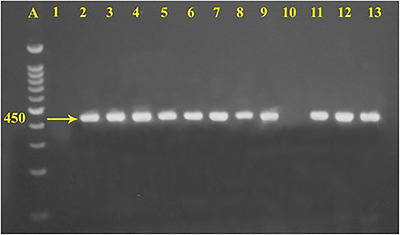
Agarose gel image of PCR products of *femA* gene. Lanes: (A) 100 bp Molecular size marker; (1) ‐ve control; (2–13) representative isolates of this study.

**FIGURE 3:**
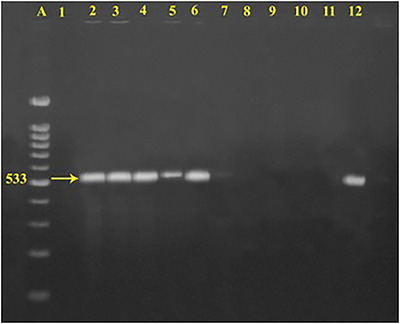
Agarose gel image of PCR products of *mecA* gene. Lanes: (A) 100 bp Molecular size marker; (1) ‐ve control; (2–12) representative isolates of this study.

PCR amplification of the *femA* gene produced the expected 450 bp amplicon. Out of the 122 biochemically confirmed *S. aureus* isolates, 107 (87.7%) were positive for the *femA* gene, and their distribution among the studied farms is shown in Table 2. Representative *femA*‐positive amplicons are shown in Figure 2. Screening for methicillin resistance revealed that nine (8.41%) of the *femA*‐positive isolates carried the *mecA* gene, indicating MRSA, as illustrated in Figure 3. The remaining 98 isolates (91.59%) were negative for the *mecA* gene.

### 3.3 *spa* Typing

**FIGURE 4:**
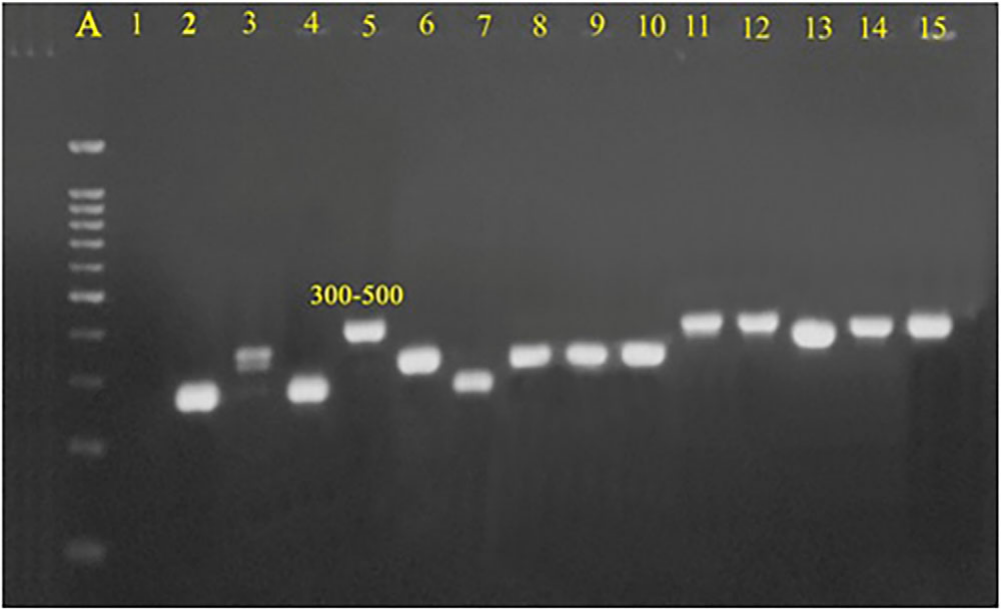
Agarose gel image of PCR products of *spa* gene. Lanes: (A) 100 bp Molecular size marker; (1) ‐ve control; (2–15) representative isolates of MRSA and MSSA from this study.

A PCR assay was performed to detect the *spa* gene in nine MRSA and one MSSA isolates. Amplicons ranging from 300 to 500 base pairs were considered positive for the *spa* gene (Figure 4). Among the nine MRSA isolates, four distinct *spa* types were identified. The most prevalent type was t011, which was found in four isolates (40%). This was followed by *spa* types t002 and t567, each present in two isolates (20%), and t1184, which was found in one isolate (10%). Additionally, the MSSA isolate was classified as *spa* type t304. Notably, *spa* types, t304, t567 and t1184, were identified for the first time in Iran (Table 3).

**TABLE 3:** Results of *spa* typing of methicillin‐resistant *Staphylococcus aureus* (MRSA) and methicillin‐sensitive *Staphylococcus aureus* (MSSA) isolated from broilers in this study.

Strain name	Resistance status	*spa* type	Countries reporting the *spa* type	Repeat ID
CC3	R	t567	European countries like Germany, Belgium, France and the Netherlands	r08‐r02‐r25‐r24‐r25
NA2	R	t567		08‐r02‐r25‐r24‐r25r
NI2	R	t1184	The Netherlands, Germany and France	r08‐r16‐r02‐r25‐r25
CD5	R	t011	European countries, mostly from Germany	r08‐r16‐r02‐r25‐r34‐r24‐r25
NG2	R	t011		r08‐r16‐r02‐r25‐r34‐r24‐r25
NE1	R	t011		r08‐r16‐r02‐r25‐r34‐r24‐r25
CF1	R	t011		r08‐r16‐r02‐r25‐r34‐r24‐r25
CL2	R	t002	Global	r26‐r23‐r17‐r34‐r17‐r20‐r17‐r12‐r17‐r16
NB2	R	t002		r26‐r23‐r17‐r34‐r17‐r20‐r17‐r12‐r17‐r16
CP4	S	t304	Global	r11‐r10‐r21‐r17‐r34‐r24‐r34‐r22‐r25

### 3.4 Antibiotic Susceptibility Test

The susceptibility of *S. aureus* isolates to the following antibiotics was determined: oxacillin, penicillin, vancomycin, cefoxitin, ceftriaxone, gentamicin, tetracycline, ampicillin, erythromycin, imipenem, clindamycin, streptomycin, doxycycline and ciprofloxacin. Resistance rates were found to be 26.17%, 85%, 0%, 24%, 38%, 55%, 52%, 87%, 54%, 0.93%, 10.28%, 12.75% and 42%, respectively.

## 4 Discussion

The present study aimed to investigate the prevalence of *S. aureus* and its antibiotic resistance patterns in poultry at a local slaughterhouse. Our findings revealed a high prevalence of *S. aureus* in both nasal and cloacal swabs, highlighting the potential for this bacterium to colonize poultry. Additionally, a significant proportion of the *S. aureus* isolates were resistant to multiple antibiotics, including methicillin, indicating the emergence of multidrug‐resistant (MDR) strains. The high prevalence of *S. aureus* in poultry could have important implications for public health. Poultry can serve as a reservoir for this pathogen, transmitted to humans through direct contact with infected animals or contaminated food products. Furthermore, the presence of MRSA in poultry poses a significant threat to human health, as these strains are often associated with severe and difficult‐to‐treat infections. The findings of this study underscore the concerning prevalence of MRSA in poultry, particularly in broiler populations. With a prevalence rate of 8.41% in broilers, our results corroborate similar findings in other global studies, establishing the poultry sector as a significant reservoir for antibiotic resistance (Nemeghaire et al. 2013; Odetokun et al. 2022). The implications of these results are far‐reaching, particularly considering the potential zoonotic transmission of MRSA, which poses considerable public health risks. The prevalence rate of MRSA reported in Algerian poultry farms, at 30%, and the flock prevalence of 27.4% in broilers, further accentuate the risks associated with MRSA in poultry production systems (Benrabia et al. 2020). Comparably, a documented prevalence of 12% in broilers in another study, with 50% of those isolates identified as MRSA, elucidates the widespread distribution of MDR strains (Bounar‐Kechih et al. 2018). Such high prevalence rates raise urgent calls for implementing control measures aimed at mitigating MRSA transmission from poultry to humans. The identification of multiple *spa* types, including novel types t304, t567 and t1184, emphasizes the genetic diversity and adaptability of MRSA strains within poultry environments (Nworie et al. 2017). The frequent detection of *spa* type t011, known for its association with livestock, raises significant concerns about environmental adaptability and potential cross‐contamination in farming conditions (Wulf et al. 2008). Such adaptability increases the likelihood of MRSA strains entering the food chain, exposing humans through consumption or direct contact with infected poultry (Aires‐de‐Sousa 2017). The predominant *spa* type, t011, identified in 40% of our MRSA isolates, aligns with its global prevalence of 3.22% across 25 countries, including Austria, Belgium, China, Denmark, France, Germany, Italy, the Netherlands and the United Kingdom (Köck et al. 2013). This type, associated with clonal complex 398 (CC398), exemplifies a livestock‐adapted lineage with zoonotic potential, frequently detected in poultry and human reservoirs, suggesting possible importation via contaminated feed or international trade (Campaña‐Burguet et al. 2025). Similarly, t002 (20% of isolates) is reported in 47 countries, including Iran, the United States, Germany and China, implying dissemination beyond local origins—potentially from migratory bird vectors or global supply chains (Asadollahi et al. 2018; Rezai et al. 2020). The novelty of t567 and t1184 in Iran (0.02% and 0.69% global frequencies, respectively) suggests local evolutionary origins, whereas t304 (MSSA, 0.69% global) bridges the human–animal interface (Hashemizadeh et al. 2019). These findings underscore the need for enhanced border surveillance in Iran's poultry sector. The predominance of t011 (40%) among the isolates is noteworthy. This *spa* type has been frequently associated with livestock, particularly in Europe, where it has been implicated in both animal and human infections. Its presence in Iran underscores the potential for cross‐border dissemination of MRSA and the importance of international collaboration in surveillance efforts (Petersen et al. 2021). The recognition of *spa* type t304 within the hospital, community and LA‐MRSA strains illustrates its diverse epidemiological landscape and suggests that geographic factors, farming practices and antibiotic usage trends play integral roles in shaping these populations (Mostofsky et al. 2011; Chen and Huang 2014). Unlike exclusively human‐adapted types, our strains’ multi‐host compatibility (evident in Ridom frequencies) signals a One Health risk, where farm‐to‐table transmission could amplify antimicrobial resistance (Hajhamed et al. 2025). Workers on poultry farms are particularly vulnerable to MRSA colonization, with evidence suggesting that close contact with infected birds and contaminated environments facilitates transmission (Manyi‐Loh and Lues 2023). A systematic review highlights that livestock workers experience a significantly higher risk of LA‐MRSA colonization compared to the general population (Chen and Wu 2021). This represents a critical area for public health intervention, as colonized workers can serve as a bridge for MRSA dissemination into broader communities, emphasizing the urgent need for improved hygiene practices and robust surveillance protocols (Krüger‐Haker et al. 2023). The extensive use of antibiotics in poultry production, which accounts for over 60% of global antibiotic production, further exacerbates the issue of antibiotic resistance (Agyare et al. 2018). This excessive use, both for therapeutic and non‐therapeutic purposes, directly contributes to the emergence of MDR strains and highlights the necessity for judicious antibiotic use alongside enhanced monitoring of NRSA in livestock to mitigate public health risks. The high resistance rates to β‐lactam antibiotics observed in this study are consistent with the *mecA*‐mediated mechanism of resistance in MRSA. The *mecA* gene encodes PBP2a, which has a low affinity for β‐lactam antibiotics, making MRSA resistant to these drugs (Lade and Kim 2023). This mechanism is a significant factor in the development of antibiotic resistance in MRSA, as it allows the bacteria to survive despite the presence of β‐lactam antibiotics (Shoaib et al. 2022). Additionally, the presence of *mecA* is often used as a marker for identifying MRSA strains in clinical settings, highlighting its critical role in resistance (Ramesh et al. 2023). Resistance to non‐β‐lactam antibiotics, such as erythromycin and tetracycline, highlights the potential co‐selection of resistance genes, possibly due to the use of these drugs in poultry farming. Studies have shown that the use of antibiotics in livestock, particularly in poultry, can lead to the emergence of MDR bacteria, including Enterobacteriaceae, which exhibit high resistance rates to both β‐lactam and non‐β‐lactam antibiotics (Amador et al. 2019). The presence of integrons and resistance genes in these bacteria indicates a significant risk of co‐selection of resistance traits, which can be exacerbated by the agricultural use of these antibiotics (Jung et al. 2022). Furthermore, the correlation between antibiotic use in farming and the spread of resistance genes underscores the need for careful monitoring and regulation of antibiotic use in agriculture to mitigate the risk of resistance development (Lade et al. 2022).

## 5 Conclusion

The findings of this study highlight the pressing need for enhanced surveillance and control measures in poultry production to address the public health risks associated with MRSA. The genetic diversity within MRSA populations necessitates continued research to understand the dynamics of resistance and to implement effective interventions. By addressing the key factors contributing to the emergence and spread of MRSA, we can better safeguard public health and minimize the impact of these MDR pathogens.

## Author Contributions

**Zeinab Alimadad**: conceptualization, methodology. **Mostafa Nemati**: data curation, writing – original draft. **Fazel Pourahmad**: software, visualization, investigation, supervision, review and editing of the manuscript.

## Funding

The authors have nothing to report.

## Ethics Statement

The work has not been previously published, except as a preprint, abstract, published lecture, academic thesis or registered report, by your policy on multiple, redundant or concurrent publication.

The manuscript is not currently being considered for publication elsewhere.

All authors have approved the manuscript for publication, and the responsible authorities where the work was conducted have granted tacit or explicit approval.

If accepted, the manuscript will not be published elsewhere in the same form, in English or any other language, including electronically, without the written consent of the copyright holder.

The authors confirm that the ethical policies of the journal, as noted on the journal's author guidelines page, have been adhered to and the appropriate ethical review committee approval has been received.

## Conflicts of Interest

The authors declare no conflicts of interest.

## Data Availability

The data supporting the findings of this study are not publicly available but can be obtained from the corresponding author upon reasonable request.
